# Assessing the Use of Different Surveillance Components to Detect Highly Pathogenic Avian Influenza Outbreaks in Poultry in the Netherlands in Low- and High-Risk Years

**DOI:** 10.1155/tbed/7441785

**Published:** 2025-01-31

**Authors:** Imke Vredenberg, Gerdien van Schaik, Francisca C. Velkers, Teun Fabri, Marcel A. H. Spierenburg, Evelien A. Germeraad, Wim H. M. van der Poel, Arjan Stegeman

**Affiliations:** ^1^Department of Population Health Sciences, Faculty of Veterinary Medicine, Utrecht University, 3584 CL, Utrecht, Netherlands; ^2^Research and Development Epidemiology, Royal GD, 7400 AA, Deventer, Netherlands; ^3^Incident and Crisis Centre, Netherlands Food and Consumer Product Safety Authority, 3511 GG, Utrecht, Netherlands; ^4^Department Virology and Molecular Biology, Wageningen Bioveterinary Research, 8221 RA, Lelystad, Netherlands

**Keywords:** active surveillance, early warning, H5N1, HPAI, passive surveillance, zoonotic

## Abstract

Avian influenza (AI) is a highly contagious zoonotic disease primarily affecting birds with clinical manifestation depending on bird species and virus subtype. Globally, outbreaks have had a large socioeconomic impact. Moreover, highly pathogenic AI virus (HPAIv) outbreaks can pose a public health risk. Detection of AIv, particularly HPAIv, mainly relies on passive surveillance, risking underreporting and delayed detection. This study describes and compares the contribution of passive and active surveillance components on HPAI detection in poultry flocks in years with different HPAIv introduction risk (free, seasonal outbreaks, and year round) in the Netherlands. We drafted a flowchart representing the flow of information and samples between farmers and veterinarians, the competent authority (CA), the national reference lab (NRL), and the private organization Royal GD and identified four different surveillance components and derived the use of each of these components during 2016 (reference), 2019 (low risk), and 2022 (high risk). The first component, “notification of suspicion,” where farmers and veterinarians directly report suspicions to the CA, accounted for 88.4% of farm visits and detected 98.1% of all HPAIv outbreaks. The second component, “testing to exclude” (TTE), consisting postmortem/sample submission and contact with the veterinary helpdesk of GD detected 2% of the cases in 2022. The third and active surveillance component, “protection zone screening,” screens farms closely to a positively detected farm. No outbreaks were detected, suggesting limited between-farm transmission. The last and active surveillance component, mandatory national serological surveillance detected two low pathogenic AI outbreaks. Analysis between years for the passive surveillance components “notification of suspicion” and “TTE,” using chi-square test of independency and odd ratios, showed increased use and farm visits in the high-risk year. However, postmortem-related submissions for TTE were increased in the disease-free year. All components combined detected HPAI or provided valuable information across different risk periods.

## 1. Introduction

Avian influenza (AI) is a highly contagious disease in birds with a variety in severity of clinical signs. The clinical manifestation depends on the pathogenicity of the virus and the bird species [1]. Low pathogenic AI virus (LPAIv) in general has mild symptoms or stays subclinical, while highly pathogenic AI virus (HPAIv) can cause high mortality rates within bird populations. The mortality is usually higher in Galliformes than in Anseriformes. LPAIv of the subtypes H5 and H7 has the potential to mutate into HPAIv [2]. AI virus is often introduced by wild birds and spreads between birds, but transmissions to mammalian species (e.g., cat, fox, cattle), including humans, has been reported [3–5]. Most of the fatal human infections with AIVs have been caused by viruses of the H7N9 and H5N1 subtypes [6, 7].

In 2005, HPAIv subtype H5N1 was introduced into Europe for the first time by wild bird migration from Asia [8]. Since 2014, HPAI H5Nx viruses have caused outbreaks in poultry in Europe each year. From 2021 onward, Europe has experienced the largest epidemic of HPAIv subtype H5N1 causing high mortality in both wild and domestic bird populations [9]. Different from previous years, the HPAIv became entrenched in resident birds, resulting in continued infections during summer [10, 11]. In total, 12,103 outbreaks, of which 4064 in domestic birds were detected in 39 countries from October 2021 until September 2023 [12, 13]. From October 2021 until October 2022, 50 million birds from commercial premises were culled in disease control in Europe [14].

The outbreaks in Europe have a large impact on animal welfare and the economy but also pose a public health risk [7, 15, 16]. Recent outbreaks on dairy farms in the United States showed again the potential of HPAI to be able to infect mammals and, this time, spillover from cattle to humans [17]. The epidemics in the Netherlands (2003, HPAIv H7N7) and France (2021–2023, HPAIv H5N1) have demonstrated the impact of HPAIv rapidly spreading between farms. During these epidemics, 255 outbreaks and 1375 outbreaks were detected, respectively [18, 19]. Early detection of HPAI outbreaks is important to prevent onward spread to other farms and countries and minimize economic impact and health risks. The current detection of HPAI outbreaks in poultry in countries where vaccination is not allowed relies on passive surveillance, in which farmers or their veterinarians report to the competent authority (CA) in case of suspicion. If HPAIv is detected, various measures are taken, including culling of infected flocks [20].

In passive, surveillance data are provided by farmers and veterinarians on their own initiative [21]. In contrast with active surveillance in which data are actively collected by the CA or other actors. Passive surveillance may suffer from under or delayed reporting because farmers do not recognize the signs of the disease or fear the consequences of a suspicion [22]. Although HPAI generally presents as a severe disease in a naïve population, in ducks signs may be less clear than in layers [23]. Consequently, adding active surveillance components may increase the effectiveness of early detection of HPAIv [24].

The aim of this study is to describe, quantify, and compare the use of multiple surveillance components for HPAI in unvaccinated poultry within three different time periods that represent differences in HPAI risk (e.g., no outbreaks, seasonal, year round) in the Netherlands. We hypothesized that the HPAI risk influenced the awareness of HPAIv suspicion and therefore expected differences in the participation in passive surveillance components between these years.

## 2. Method

### 2.1. Time Period

For this study, the calendar years 2016, 2019, and 2022 were chosen, as these years differed regarding the epidemiological manifestation of HPAIv. The outbreak of 2016 was chosen because of the seasonal manifestation. Within the last 3 months of 2016, nine outbreaks of HPAIv in commercial poultry farms were notified. There were no other holders with >50 animals reported positive. The year 2019 was chosen because no outbreaks of HPAIv were notified in the Netherlands. Finally, the year 2022 was included because of the 99 outbreaks of HPAI year round on commercial farms and holders with >50 animals [25].

### 2.2. Detection Pathways

In the Dutch surveillance system, there are several components that can lead to the detection of AIv and more specifically HPAIv. After an inventory using literature and conversations with experts, five main actors were identified: farmers, veterinarians, the Netherlands Food and Consumer Product Safety Authority (NVWA) further referred to as the CA, Wageningen Bioveterinary Research (WBVR) further referred to as national reference lab (NRL), and the animal health service, Royal GD (GD), a private organization that is commissioned the voluntary national animal health surveillance program by the government and poultry sector [26–28]. Source of information, information flow, sampling procedures, and shared results on HPAI-related surveillance components were discussed with experts from the CA, NRL, and GD. The expert information, combined with literature, resulted in a flowchart representing the relation between the actors within the four identified surveillance components, that is, (1) notification of suspicion based on clinical signs, (2) testing to exclude (TTE), which includes sample testing and postmortem examination, (3) protection zone screening, and (4) national serological surveillance [29]. The activities within the surveillance components were quantified within the three different time periods for commercial farms and holders with >50 animals. Surveillance of wild birds was not included in this study. The flowchart was constructed using Adobe Illustrator version 27.4.1.

### 2.3. Available Data

Two different data sources were used to quantify the activities for each surveillance component depicted in the flowchart: the annual surveillance reports on poultry health of GD and a dataset of the CA, both for the years 2016, 2019, and 2022 [25]. The data shown in the results were directly copied or calculated from these reports. The number of AI-related contacts of the helpdesk were provided by GD. The total number of poultry farms originates from Statistics Netherlands [30].

Notifications of a suspicion by a veterinarian or farmer to the CA that did not result in a farm visit were not reported in the yearly reports. These data were, however, supplied by the CA and contained farm ID, date, case ID, method (e.g., clinical, monitoring, screening), and result (e.g., no action, negative, positive, positive H5N8 or H5N1). Aggregating by case ID prevented double counts. The column “method” described the origin of the record. “Clinical” was assumed to be direct contact between farmers or veterinarians and the CA. “Monitoring” was related to the national serological surveillance for AI. “Screening” was assigned to farms tested because they were located within 3 km of an outbreak. If “no action” was reported as result no follow-up of any sort (e.g., test, farm visit) was conducted. For five outbreaks in 2022, the GD year report and the CA data differed in the attribution of these outbreaks to the surveillance components of protection zone screening and notification of suspicion. After corresponding with the CA and GD, the attribution of the CA was assumed to be correct.

### 2.4. Data Analysis

After the quantification of the surveillance components, the differences between years were analyzed for notification of suspicion and TTE using the chi-square test of independency. Odd ratios and 95% confidence intervals were calculated to further analyze differences between years. In all cases, 2016 was used as the reference year. Reported suspicions and submitted samples can prove positive or negative for HPAIv. The number of positive findings is a function of both the incidence of HPAIv introductions and the awareness of the farmers and veterinarians. Because the first varies from year to year, the ratio of the number of positive findings and the number of farms present cannot be used as a measure of awareness. However, assuming that other diseases that would be part of the differential diagnosis of notifiable HPAI would be constant over the years, the ratio between the number of negative findings and the total number of farms present was considered as a measure of awareness. Under that assumption, we tested whether the ratio between suspicions/submissions that turned out negative and the total number of farms minus the HPAIv positive ones was the same across the 3 years. For the notification of the suspicion component, the ratio of contact resulting in a farm visit and the ratio of farm visits resulting in detection of HPAIv were analyzed as measure of the awareness of the CA. We used the Fisher's exact test for the ratio of the positive farm visits. For TTE, we compared the number of samples sent to the NRL by GD with the number of postmortem examinations which did not result in the submission of samples. The data were analyzed using R version 4.2.2 [31]. We used the “CrossTable” function from the R-package “gmodels” for the chi-square test and the function “oddsratio” of the R-package “epitools” to calculate the odd ratios and their confidence intervals [32, 33].

## 3. Results

### 3.1. Composition

Table 1 shows the composition of the Dutch poultry sector across the studied years. In general, the number of poultry farms decreased by 260 farms, the total number of animals also decreased, but the average farm size increased between 2016 and 2019. The two main subsectors are layers (mean = 967 farms) and broilers (mean = 873 farms). The number of turkey and duck farms decreased by 10 over the years. The number of animals in miscellaneous (e.g., guinea fowl, quail, geese) increased in 2019 sixfold (*n* = 201,900 birds); but in 2022, the number dropped back to the level of 2016 (*n* = 32,100 birds).

### 3.2. Surveillance Components

**Figure 1:**
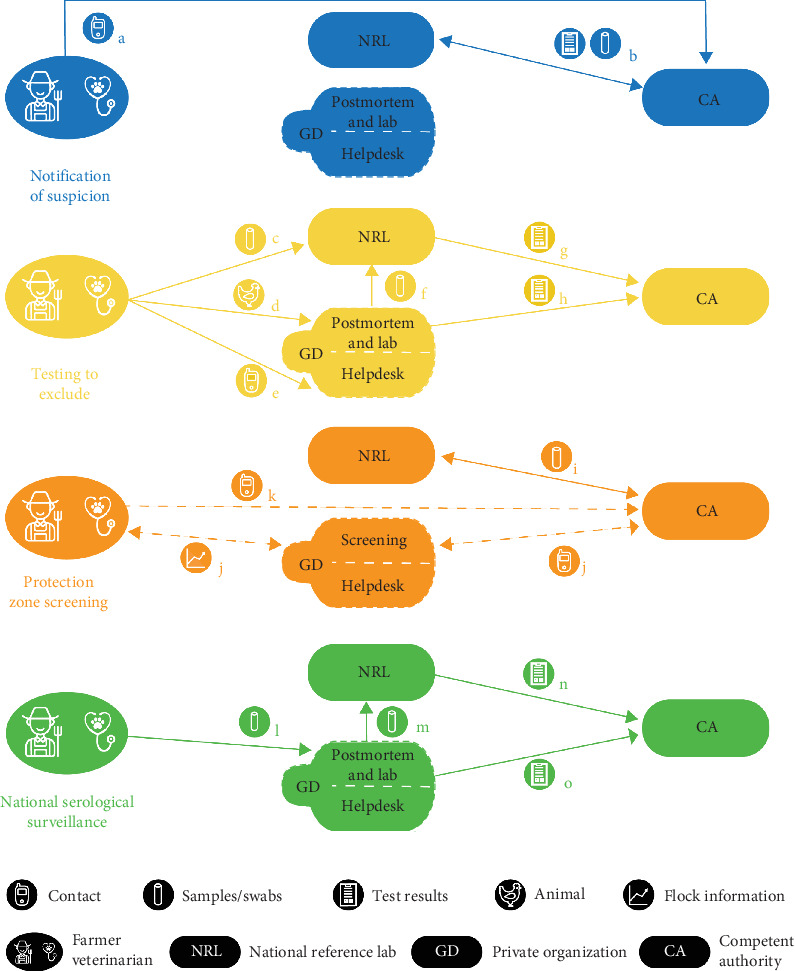
Visualization of the information and sample flows of the surveillance components on early detection of AI virus in the Netherlands. The letters a–o represent the origin and direction of contact (phone icon), samples (test-tubes icon), test results (clipboard icon), animal (chicken icon), or flock information (graph icon) and correspond with the letters of Table 2. The other icons represent farmers and veterinarians, the national reference lab (NRL), private organization GD, and the competent authority (CA).

Figure 1 shows the four different surveillance components (suspicion, exclusion, screening, serology), which can lead to the detection of an HPAI outbreak in the Netherlands.  Table 2 shows the numbers of animal submissions (chicken icon), swab samples submissions (test-tubes icon), contacts (phone icon), positive results (clipboard icon), or farms included in data screening (graph icon) of which the letters a–o correspond to the pathways within the four components. Farms can be included in more than one component at the same time. For example, samples may have been submitted for TTE because at the time of submission clinical signs were not expected to be caused by HPAIv infection. When signs change or a threshold (see below) is reached before the TTE result is known, suspicion will be reported to the CA and the farm will be in two components at the same time.

### 3.3. Notification of Suspicion

The component suspicion (blue in Figure 1) shows the direct reporting of farmers and veterinarians to the CA in case of suspicion of HPAI on the farm (e.g., severe clinical problems). These reports are obligatory in case of an increased mortality of 0.5% on two consecutive days from 10 days of age for broilers and layers, 1% for turkeys within two consecutive days, and 3% within a week for all poultry. In addition, the veterinarian is to be contacted if water and feed consumption or egg production drops by 5% or more for two consecutive days [23, 29]. In 2022, the rules were tightened due to the size of the outbreak. In addition to the rules described above, farmers also had to report if mortality was tripled for 2 days compared to the average mortality of last week for broilers and layers. For ducks farmers, it became obligatory to report if after 7 days of age, the mortality was 0.15% on two consecutive days or 0.5% mortality simultaneously with a decrease of 5% food intake on 1 day [34]. In case of suspicion of HPAIv infection by the farmer or veterinarian, direct reporting should follow. Subsequently, an official CA veterinarian, accompanied by a veterinarian of GD and the practitioner, visit the farm and takes 20 trachea and 20 cloaca swab samples per flock. Swabs are tested by PCR at the NRL in pools of 5 and blood samples are tested for antibodies by viral nucleoprotein antibody ELISA, followed by an HI test in case of positive findings [35].

Both, in 2016 and 2019, 40 suspicions were reported to the CA, resulting in 21 farm visits in 2016 and six in 2019 (Table 2). In 2016, nine positive HPAI outbreaks were confirmed; in 2019, all samples proved negative. In 2022, 185 contacts resulted in 126 farm visits and 97 farms were found positive for HPAIv. Although, the proportion of farms that were detected positive for HPAIv in 2022 was higher, the proportions of reported suspicions resulting in a farm visit were not significantly different between 2016 and 2022. In contrast, in 2019, the proportion of suspicions that resulted in a farm visit by CA was significantly lower compared to 2016.

### 3.4. TTE

The exclusion component (yellow in Figure 1) is used when clinical signs are present but unlikely to be associated with HPAI according to the veterinarian of the farm and thresholds are not exceeded (mortality, food intake, water intake, egg production). Samples are submitted by the veterinarian to confirm the absence of AI virus. Different pathways can be followed to exclude HPAIv as the pathogen causing the clinical signs; swab samples can be directly submitted to the NRL, animals can be submitted to GD for postmortem examination or the situation can be discussed with the GD veterinary helpdesk [36]. In case of postmortem examination, the pathologist can submit samples of the birds for AIv testing to the NRL if he or she considers this appropriate. Positive results of TTE samples analyzed by the NRL and suspicion raised by the pathologist upon postmortem examination will be reported to the CA. In both situations, the CA will visit the farm to take official samples to confirm if the farm is officially positive for HPAI. If the GD helpdesk suspects AI during a contact, they will advise the farmer or veterinarian to report a suspicion to CA; according to Dutch law, it is the obligation of the farmer/veterinarian to report a suspicion to the CA in that situation.

In 2016, 2019, and 2022, 174, 159, and 354 submissions were sent directly to the NRL. In 2022, there were significantly more TTE submissions of samples from veterinarians to the NRL compared to 2016. Farmers and their veterinarians sent 752, 826, and 413 submissions for postmortem to GD in 2016, 2019, and 2022, respectively, resulting in 211, 280, and 126 samples submitted by GD to the NRL. Submissions to postmortem dropped markedly in 2022 when compared with the other years. In addition, in these years, GD received 24, 87, and 1 AI-related contacts at the helpdesk. The proportion of contact with the helpdesk was significantly different across the years.

While no positive farms were found in 2016 and 2019, two farms were detected positive in 2022. The first one is after the submission of samples by a veterinarian to the NRL. For the second one, samples were taken and sent to the NRL, but the suspicion was already reported after postmortem findings.

### 3.5. Protection Zone Screening

The protection zone screening (orange in Figure 1) is an active surveillance component which is initiated by the CA. If a positive farm is detected, in a 3 km zone, every poultry farm is visited as part of the screening procedure and 20 trachea and 20 cloaca swab samples are taken per house for RT-PCR [37]. Depending on the estimated risk in the area, birds on poultry farms within a 1 km zone are culled in high-risk areas or selected for 2 weeks of remote screening by GD in low-risk areas [38]. The remote screening was only applicable to the year 2022 and consists of the exchange of flock data like mortality, egg production, food, and water intake which will be compared to a reference day. In case of decreases in production and food or water intake raising suspicion of AI, GD will notify the CA. Independent from the remote screening, the farmer or the veterinarian has to notify the CA if they suspect an infection of AI.

In 2016, 2019, and 2022, samples for screening were taken by the CA 25, 6, and 286 times, respectively. Only in 2016, screening on samples resulted in one farm visit with a negative result for HPAIv. In 2022, farms were also remotely screened. The remote screening was performed on 266 farms and resulted in two farm visits. These two farms were also found to be negative for HPAI.

### 3.6. National Serological Surveillance

The serology component (green in Figure 1) represents the national serological surveillance which is obligatory for all Dutch poultry farms with the aim of detecting AIv in general [29]. Farms are sampled yearly for the presence of antibodies against AIv. Free-range poultry are sampled four times a year, and other types once a year. Every sampling round, 30 blood samples are taken of which the distribution between houses on the farm should be equal [35]. The samples are analyzed with NP-ELISA by GD and seropositive tests are sent to the NRL for confirmation by performing NP-ELISA followed by HAR in case of seropositive samples. If 30% or more of the samples of a submission are seropositive for AIv at GD, this leads to direct notification to the CA by GD and all samples of the submission will be send to the NRL. If samples are positive at NRL, latter will notify the CA, resulting in a farm visit.

In 2016, 2019, and 2022, GD received 3394, 3641, and 3383 submissions (30 samples per submission) for serology, respectively. Although this resulted in 10 and 8 farm visits in 2016 and 2019, no HPAIv was detected. In 2016, two farms were detected positive for LPAIv subtype H5 or H7. The percentage of submissions with more than 30% positive samples at GD differed between the years. In 2022, there were no positive serology results and, consequently, no farm visits.

### 3.7. Total of Surveillance Components

In total, 33, 14, and 126 farm visits were performed in the years 2016, 2019, and 2022, respectively. These years differed in absolute numbers of visits but also differed in the percentage of positive HPAIv cases detected based on these farm visits. In 2022, 78.6% of the farm visits resulted in HPAIv detection, which was much higher compared to 27% in 2016. In total, 63% of the farm visits resulted in the detection of HPAIv of which 98% was detected by notification of suspicion by farmer or veterinarian.

### 3.8. Comparing the Use of Surveillance Component Across the Years

To correct for differences in HPAI exposure across the years, we excluded the suspicions that resulted in the detection of an outbreak and showed that there was a significant difference between the number of reported suspicions across years (*χ*^2^ [2, *N* = 5714] = 50.79, *p*  < 0.001). Even though no outbreaks were confirmed in 2019, the number of reported suspicions was not significantly different in 2019, compared to 2016 (Table 3). In the year 2022, significantly more suspicions were reported than in 2016, with an increased odds of 3.58 (2.39–5.51). Also, the use of TTE submissions by farmers and veterinarians directly to the NRL was significantly different across years (*χ*^2^ [2, *N* = 5818] = 140.6, *p*  < 0.001) with an increased odds of 2.67 (2.20–3.25) in 2022 compared to 2016. In 2019, farmers were more likely (OR = 1.40, [1.23–1.59]) to submit poultry for postmortems compared to 2016. Surprisingly, farmers were less likely (OR = 0.53, [0.46–0.61]) to submit for postmortem in 2022. When examining the proportion of reported suspicions resulting in official fam visits by CA, in 2019, the odds of visiting farms after CA had been contacted were lower than in 2016 (OR = 0.16, [0.05–0.46]) (*χ*^2^ [2, *N* = 265] = 38.5, *p*  < 0.01). In 2022, visited farms had an increased odds of being positive for HPAIv compared to 2016, (OR = 6.64, [2.46–18.72]), although the likelihood of a farm visit upon a reported suspicion was not significantly different across both years. Last, GD had an increased odds of submitting samples from postmortems to the NRL in 2019 compared to 2016 (OR = 1.31, [1.06–1.63]) (*χ*^2^ [2, *N* = 1991] = 6.33, *p*=0.04). In summary, 2022 differed significantly from 2016 for all elements except the submissions from GD to the NRL for TTE, which was, like postmortems to GD, significantly higher in 2019.

## 4. Discussion

This study described, quantified, and compared four surveillance components (notification of suspicion, TTE, protection zone screening, and national serological surveillance) for detection of HPAIv in 3 years representing different risk levels (no outbreak, seasonal, year round) in the Netherlands. Five actors were involved in the surveillance components, the farmers and veterinarians providing the observation of clinical signs, samples, animals, or other farm information, the NRL, GD with a helpdesk, postmortem, and diagnostic laboratory in service of the national animal health surveillance program, and the CA.

The contribution of the indicated components differed between the years, but all detections of HPAIv were found through the passive surveillance components. Between years, 2022 (year round risk) had a higher percentage of submitted samples to the NRL for TTE but was lower in helpdesk contact and postmortems. All years differed in the number of helpdesk contacts received at GD and reporting's of GD to the CA based on serological surveillance. The total number and percentage of detected HPAIv-infected farms confirmed that the years differed in epidemiological manifestation, as we intended in this study.

The awareness of farmers and veterinarians, the GD, and CA was tested within several comparisons, with 2016 (seasonal) as reference year. Assuming that the occurrence of other poultry diseases was equal over the years, differences in contacts or action rates between years, when excluding outbreaks, would not have been expected if the awareness between years was equal. Nevertheless, the ratio of farmers and veterinarians reporting to the CA and submitting samples to the NRL increased, and therefore the awareness of farmers was increased in 2022. In time of a continuous HPAI circulation as was the case in 2022, they seemed to opt for direct reporting and submissions of samples and leaned toward postmortem in the absence of HPAI. The high mortality of HPAI, visitor prohibition measures on farms, and a transport ban of two times 4 weeks in the area of the GD facility, probably also influenced the postmortem submissions. In 2019, the CA did less farm visits compared to 2016, but this year no HPAIv was found, unlike in 2016. Although this was not considered concerning as the disease was not present in 2019, it suggests the decision of CA to visit depends on the epidemiological situation. In 2016 and 2022, farmers and veterinarians reported more suspicious cases to the CA or submitted samples directly to the NRL resulting in less submission of samples by GD to the NRL in 2016 and 2022, compared to 2019. The awareness of farmers, veterinarians, and the CA seemed increased during outbreaks. The awareness of GD based on postmortems was almost constant but somewhat higher in the year without outbreaks (2019).

Notification of suspicion to the CA resulted in the vast majority of detections of HPAIv, which demonstrated the importance of passive surveillance. Legal obligations, changing social norms, and limited negative effects around false suspicions likely contributed to the willingness of farmers to report suspected cases [22]. For example, improved technology such as PCR-based diagnostics enables quick lifting of isolation of the farm within 8–12 h in case tests turn out negative. The effectiveness may also be explained by the financial compensation farmers receive for every animal alive when HPAIv is confirmed. Early recognition and reporting of HPAI can therefore lead to higher financial compensation. The compensation is paid by the National Animal Health Fund. Farmers, government, and the EU contribute to this fund [39].

The TTE component is put in place to detect all kinds of poultry diseases and monitor health trends and is not restricted to AIv. In 2022, TTE resulted in detection of two HPAIv outbreaks, which is 2.0% of all outbreaks in 2022. One farm was confirmed positive by the NRL after submission of a sample of a postmortem by GD, the other was submitted directly from a veterinarian to the NRL. In these two cases, the clinical manifistation might have been different from what was expected by the farmer and veterinarian, leading to the decision to not directly report a HPAI suspicion. How much later these positive cases would otherwise have been detected by other components remains unknown. Missed or delayed detection of HPAIv outbreaks can increase animal and public health and economic risks [16, 18, 19]. In other years, TTE did not result in detection of the virus indicating that the other surveillance components were effective in disease detection. Demonstrating that TTE is primarily used to exclude HPAIv as disease cause. This combined with the high number for notification of suspicion strengthens the argument that there is no reluctance to directly report to the CA in the Netherlands in the case of HPAI.

Over the years, the number of helpdesk contacts differed. The 87 contacts of 2019 consisted of contacts about serological results, but distinguishing between contacts on these results and contacts on clinical symptoms was not possible. However, comparing the numbers of the helpdesk with the serological surveillance, and especially the number of reports of GD to the CA (Table 2, letter o), it indicates that also in the other years, the contacts were mainly about results of the serological surveillance. Besides the registered contacts, personal contact or by app is not registered probably resulting in an underestimation of the contact.

The serological surveillance did not detect HPAIv in any year but did detect LPAIv in 2016. Due to the clinical manifestation and high case fatality, HPAI will normally be detected before the serological samples are tested. It is a more relevant tool to detect LPAIv as shown in 2016. The reason no LPAIv was detected in 2022 is most likely caused by the housing order that was in effect for most of that year and that was demonstrated to reduce LPAIv introductions [40].

Like serological surveillance, protection zone screening did not detect any HPAIv infections. This indicated the absence of between farm spread in the protection zone in the three studied years and suggests timely outbreak detection. Negative results from neigbhoring farms can prevent culling and provide information about virus transmission between farms or external factors such as wild birds or other ways of contamination. The screening of samples and the screening of farm data might also increase the awareness of farmers to the importance of their biosecurity measures and to monitor mortality rates, production rates, and food and water intake of their flocks.

In this study, we used calendar years and did, therefore, not cover the whole seasonal outbreak of 2016/2017, which continued in the first months of 2017, because of the data availability in the form of annual reports. An advantage of using calendar years is that both outbreaks and use of the surveillance components are quantified over a full year and thus comparable. The start and finish of an outbreak season can only be established with hindsight, can greatly vary in length, and will not necessarily match the perceived risk period. We think that the different calendar years sufficiently reflected the epidemiological context and the risk perception for HPAI. There was a clear difference between the different pathways in different disease status situations, as was intended by the choice of the years included, and the procedure was equal for each year.

The data originates from yearly reports of GD, the raw data were not available for privacy reasons. The data of the CA were used to find the number of contacts for the suspicion component. It is still possible that farmers or veterinarians first contacted the veterinary helpdesk and reported the suspicion to the CA later on. We were not able to extract these contacts and assign them to the TTE component. Therefore, there might be an underestimation of the veterinary helpdesk contribution. However, this effect is expected to be small. As we described earlier, most contacts are expected to be about results of the serological screening rather than HPAI-related clinical signs, which was confirmed by the helpdesk veterinarians.

This analysis focused on the Dutch surveillance system for HPAI in unvaccinated poultry and shows that differences in contribution between components can be found between years of different epidemiological manifestation of HPAI using empirical data. However, surveillance systems and vaccination status of poultry differ between countries. Available resources, cultural differences, trust in executional authorities, and economic motivations of farmers and veterinarians should be taken into account translating these results to other countries. For instance, live bird markets in parts of Asia and Africa (e.g., Bangladesh, Vietnam, Nigeria) and the prevalence of free-range poultry (e.g., China) increase the risk of disease spread, and the use of vaccination, may require different or additional surveillance components or different usage of comparable components [41–44]. The United States, for example, has a more comparable system that compensates poultry alive at the moment of HPAI confirmation [45]. This study can serve as an example for countries that want to develop, adapt, or evaluate their own surveillance systems for HPAI.

The comparison of the effectiveness of different components within years that differed in HPAI risk status was the main purpose of the study. Therefore, we chose three specific years based on the epidemiological manifestation of HPAI in those years, instead of a serie of consecutive years. The results give insight and show clear differences in the effectiveness of surveillance components between years of different risk levels (freedom of disease, seasonal oubreaks, year round). Comparing multiple years of the same risk level can give more information on the generalizability of the results to other years, but this was beyond the scope of this study.

Although this study focused on HPAI, the Dutch surveillance system is designed to early detect outbreaks of (re)emerging and exotic diseases. In 2020, this surveillance system also detected the SARS-CoV-2 outbreak in mink [46]. The early detection of mink and cooperation between the stakeholders efficiently controlled the outbreak [47]. As shown in mink, a good-performing surveillance system can be crucial in detecting and controlling zoonotic diseases. Evaluation of the system is important to become aware of the systems strengths and weaknesses, which makes improvement of the system possible.

Every component in place adds to effectiveness in the early detection of HPAIv or provides information on the disease presence of AIv in general. Besides the effectiveness in disease detection, a cost-effectiveness analysis can provide insight in the relationship between the effectiveness of HPAIv detection and financial costs and benefits. This can help policymaker in taking evidence-based decisions on the added value of each HPAI surveillance components. As the passive surveillance components target not only HPAI but also other emerging disease, cost-effectiveness analysis is complex and beyond the scope of this study. Further investigation into the cost-effectiveness is recommended.

## 5. Conclusion

Four different surveillance components were involved in the detection of HPAIv in 2016, 2019, and 2022 in the Netherlands. These years were different from each other in epidemiological manifestation of HPAI. The passive surveillance component notification of suspicion, consisting of direct reporting of HPAI to the authorities, detected 98.1% of the HPAIv infections and the component used for exclusion diagnostics the remaining 1.9%. The serological surveillance and protection zone screening with sampling of farms, within 3 km distance of an infected farm, did not result in HPAI detection. All components combined, accurately detected HPAIv, or provided valuable information on the HPAIv outbreaks. Although, postmortems for TTE were executed more often within the HPAIv-free year, the awareness of farmers, veterinarians, and CA was increased in years of disease presence.

## Figures and Tables

**Table 1:** Demography of poultry farms in the Netherlands over the years 2016, 2019, and 2022 [30].

Farmtype	2016			2019			2022		
	Farms	Animals (×1000)	Farm size	Farms	Animals (×1000)	Farm size	Farms	Animals (×1000)	Farm size
Layers	1090	47,689	43,752	920	45,894	49,884	890	44,287	49,760
Broilers	910	57,930	63,659	870	55,848	64,192	840	53,246	63,388
Turkey	40	762	19,050	30	532	17,720	30	576	19,187
Ducks	60	931	15,510	50	968	19,360	50	644	12,882
Miscellaneous^a^	10	33	3260	20	202	10,095	10	32	3210
Total	1970	107,345	50,874	1770	103,444	54,732	1710	98,785	54,277

^a^For example: guinea fowl, quail, and geese.

**Table 2:** Quantification of the surveillance components on early detection of avian influenza virus (AIv) in the Netherlands.

Path	Component	2016		2019		2022	
	Notification of suspicion	Total (*n*)	%	Total (*n*)	%	Total (*n*)	%
a	Contact CA	40	—	40	—	185	—
b	Farm visits and swabs	21	52.5	6	15.0	126	68.1
	Positive HPAIv	9	22.5	0	0	97	49.7
	Testing to exclude	—	—	—	—	—	—

	Total c + d + e	950	—	1072	—	768	—
c	Submission to NRL	174	18.3	159	14.8	354	46.1
d	Submission postmortem to GD	752	79.2	826	77.1	413	53.8
e	Contact helpdesk GD	24	2.5	87	8.1	1	0.1
f	Samples GD to NRL	211	22.2	280	26.1	126	16.4
g	Positive results NRL to CA	1	0.1	0	0	3	0.3
h	Positive postmortem GD to CA	0	0	0	0	1	0.1
	Farm visits	1	0.1	0	0	3	0.3
	Positive HPAIv	0	0	0	0	2	0.3
	Protection zone screening	—	—	—	—	—	—

	Total i + j	—	—	—	—	552	—
i	Sample screening	25	—	6	—	286	51.8
j	Submissions swabs NRL	25	100	6	100	286	51.8
k	Farms in screening on indicators	NA	NA	NA	NA	266	48.2
	Farm visits	1	4.0	0	0	2	0
	Positive HPAIv	0	0	0	0	0	0

	Serological surveillance	—	—	—	—	—	—
l	Submission serology	3394	—	3641	—	3383	—
m	Samples to NRL	351	10.3	409	11.2	326	9.6
n	Results NRL to CA	14	0.4	17	0.5	0	0
o	Results GD to CA	27	0.8	91	2.4	2	<0.1
	Farm visits	10	0.3	8	0.2	0	0
	Positive HPAIv	0	0	0	0	0	0

	Total						

	Total farm visits	33	—	14	—	126	—
	Total HPAIv positive	9	27	0	0	99	78.6
	Total LPAIv positive	2	6	0	0	0	0

*Note:* The letters in the first column correspond with Figure 1, which visualizes the components. The component column shortly describes the pathway within the surveillance component. Percentages are calculated from the total (top row) of each component. Summations of pathways are presented by the summation of the letters. HPAIv is highly pathogenic, and LPAIv is low pathogenic avian influenza virus, GD is Royal GD.

Abbreviations: CA, competent authority; NRL, national reference laboratory.

**Table 3:** Comparison of the level of usage of stakeholders in 3 years with different risk levels (free = 2019, seasonal = 2016, endemic = 2022) regarding the Dutch surveillance components to detect highly pathogenic avian influenza virus (HPAIv).

Awareness stakeholders	2016	2019		2022	
		OR	95% CI	OR	95% CI
Farmers and veterinarians^b^					
Notification of suspicion to CA	Reference	1.44	0.90–2.33	3.58^a^	2.39–5.51
Sample submissions NRL	Reference	1.02	0.82–1.28	2.67^a^	2.20–3.25
Postmortem submissions GD	Reference	1.40^a^	1.23–1.59	0.53^a^	0.46–0.61
Competent authority					
Farm visits out of contact CA	Reference	0.16^a^	0.05–0.46	1.92	0.95–3.88
Farm visits detecting HPAI positive farms	Reference	NA	NA	6.64^a^	2.46–18.72
Royal GD					
Samples of postmortems send to the NRL	Reference	1.31^a^	1.06–1.63	1.13	0.86–1.46

*Note:* GD is Royal GD.

Abbreviations: CA, competent authority; NRL, national reference laboratory.

^a^Significantly different compared to 2016.

^b^Comparison with total farm population.

## Data Availability

The annual reports are available online on the website of Royal GD or by request. The data of the Netherlands Food and Consumer Product Safety Authority (NVWA) are for privacy reasons only available upon request.
